# Supplementary material for: No evidence of host-specific egg mimicry in Asian koels
Source: PLoS One. 2021 Jul 9;16(7):e0253985. doi: 10.1371/journal.pone.0253985 (PMC8270166; doi:10.1371/journal.pone.0253985)
Supplement: S1 Table — Asian koel eggs and host eggs were assessed separately, and host egg were accounted for non-independence. AK_CM = Asian koel in common myna nests, AK_HC = Asian koel in house crow nests and AK_LTS = Asian koel in long-tailed shrike nests. (DOCX) [file pone.0253985.s001.docx]

## Supplementary Material

**Table S1: Linear mixed effects model using log-transformed egg characteristics as response (log(volume), log(shape) and log pattern variables), for Asian koel eggs and three host species (common myna, house crow and long-tailed shrike). Asian koel eggs and host eggs were assessed separately, and host egg were accounted for non-independence. AK_CM = Asian koel in common myna nests, AK_HC = Asian koel in house crow nests and AK_LTS = Asian koel in long-tailed shrike nests.**

| **Egg type** | **Egg character** | **Estimate** | **SE** | ***z*** | ***P*** |  |
| --- | --- | --- | --- | --- | --- | --- |
| AK_CM-AK_HC | Volume | -0.01 | 0.03 | -0.20 | 0.803 |  |
| AK_CM-AK_LTS | Volume | 0.06 | 0.02 | 3.70 | <0.001 | *** |
| AK_HC-AK_LTS | Volume | 0.07 | 0.03 | 2.4 | 0.015 | * |
| Common Myna-House Crow^1^ | Volume | 0.76 | 0.01 | 56.00 | <0.001 | *** |
| Common Myna-Long-tailed Shrike^1^ | Volume | -0.46 | 0.02 | -29.50 | <0.001 | *** |
| House Crow-Long-tailed Shrike^1^ | Volume | -1.22 | 0.02 | -73.00 | <0.001 | *** |
| AK_CM-AK_HC | Shape | -0.03 | 0.01 | -2.58 | 0.010 | * |
| AK_CM-AK_LTS | Shape | -0.01 | 0.01 | -1.19 | 0.236 |  |
| AK_HC-AK_LTS | Shape | 0.02 | 0.01 | 1.80 | 0.072 |  |
| Common Myna-House Crow | Shape | 0.03 | 0.01 | 4.68 | <0.001 | *** |
| Common Myna-Long-tailed Shrike | Shape | -0.09 | 0.01 | -11.76 | <0.001 | *** |
| House Crow-Long-tailed Shrike | Shape | -0.11 | 0.01 | -14.61 | <0.001 | *** |
| AK_CM-AK_HC | Max energy | 0.24 | 0.12 | 1.99 | 0.046 | *^2^ |
| AK_CM-AK_LTS | Max energy | 0.01 | 0.11 | 0.07 | 0.947 |  |
| AK_HC-AK_LTS | Max energy | -0.23 | 0.15 | -1.57 | 0.117 |  |
| House Crow-Long-tailed Shrike | Max energy | 0.42 | 0.08 | 5.12 | <0.001 | *** |
| AK_CM-AK_HC | Prop energy | 0.05 | 0.04 | 1.50 | 0.134 |  |
| AK_CM-AK_LTS | Prop energy | -0.00 | 0.03 | 0.00 | 0.998 |  |
| AK_HC-AK_LTS | Prop energy | -0.05 | 0.04 | -1.48 | 0.140 |  |
| House Crow-Long-tailed Shrike^1^ | Prop energy | 0.20 | 0.04 | 5.65 | <0.001 | *** |
| AK_CM-AK_HC | Sum energy | 0.18 | 0.11 | 1.63 | 0.103 |  |
| AK_CM-AK_LTS | Sum energy | 0.04 | 0.10 | 0.37 | 0.714 |  |
| AK_HC-AK_LTS | Sum energy | -0.14 | 0.14 | -1.05 | 0.294 |  |
| House Crow-Long-tailed Shrike | Sum energy | 0.26 | 0.08 | 3.41 | <0.001 | *** |
| AK_CM-AK_HC | SD energy | 0.17 | 0.12 | 1.45 | 0.148 |  |
| AK_CM-AK_LTS | SD energy | -0.11 | 0.09 | -1.20 | 0.230 |  |
| AK_HC-AK_LTS | SD energy | -0.28 | 0.12 | -2.29 | 0.022 | *^2^ |
| House Crow-Long-tailed Shrike | SD energy | 0.46 | 0.08 | 5.64 | <0.001 | *** |
| ^1^Residuals marginally deviating from normal distribution.  ^2^Significant when log-transformed only. | | | | | | |
