# Supplementary material for: No evidence of host-specific egg mimicry in Asian koels
Source: PLoS One. 2021 Jul 9;16(7):e0253985. doi: 10.1371/journal.pone.0253985 (PMC8270166; doi:10.1371/journal.pone.0253985)
Supplement: S2 Table — Each egg characteristic was used as response, and koel vs. host as a fixed factor. (DOCX) [file pone.0253985.s002.docx]

## Supplementary Material

**Table S2: Model outputs from linear mixed-effects models showing differences between Asian koel eggs from specific host nests and their corresponding host eggs (see Figs 2 and 3). Each egg characteristic was used as response, and koel vs. host as a fixed factor.**

| **Egg character** | **Asian koel vs host** | ***n***  ***eggs tot*** | ***n clutches*** | ***n***  ***koel eggs*** | ***Estimate host difference*** | ***z*** | ***P*** |  | |
| --- | --- | --- | --- | --- | --- | --- | --- | --- | --- |
| Volume^1^ | Common Myna | 556 | 295 | 167 | -1886.4±120.20 | -15.700 | < 0.001 | *** | |
| Volume | House Crow | 326 | 121 | 22 | 5727.5±437.20 | 13.100 | < 0.001 | *** | |
| Volume^1^ | Long-tailed Shrike | 294 | 141 | 82 | -5291.0±117.70 | -44.970 | < 0.001 | *** | |
| Shape | Common Myna | 556 | 295 | 167 | 0.03±0.01 | 3.890 | < 0.001 | *** | |
| Shape^1^ | House Crow | 326 | 121 | 22 | 0.11±0.02 | 5.960 | < 0.001 | *** | |
| Shape^1^ | Long-tailed Shrike | 294 | 141 | 82 | -0.06±0.01 | -6.820 | < 0.001 | *** | |
| Max Energy | House Crow | 122 | 47 | 12 | 748.14±93.37 | 8.013 | < 0.001 | *** | |
| Max Energy^1^ | Long-tailed Shrike | 90 | 49 | 32 | 1443.00±135.40 | 10.656 | < 0.001 | *** | |
| Prop Energy^1^ | House Crow | 122 | 47 | 12 | 0.00±0.01 | -0.737 | 0.461 |  | |
| Prop Energy | Long-tailed Shrike | 90 | 49 | 32 | 0.03±0.00 | 7.070 | < 0.001 | *** | |
| Sum Energy | House Crow | 122 | 47 | 12 | 7474.00±861.20 | 8.679 | < 0.001 | *** | |
| Sum Energy^1^ | Long-tailed Shrike | 90 | 49 | 32 | 10435.00±1091.00 | 9.566 | < 0.001 | *** | |
| SD Energy | House Crow | 122 | 47 | 12 | 199.07±24.52 | 8.119 | < 0.001 | *** | |
| SD Energy^1^ | Long-tailed Shrike | 90 | 49 | 32 | 392.90±37.41 | 10.503 | < 0.001 | *** | |
|  | ^1^Residuals deviating from normal distribution. | | | | | | | |  |
