# Supplementary material for: No evidence of host-specific egg mimicry in Asian koels
Source: PLoS One. 2021 Jul 9;16(7):e0253985. doi: 10.1371/journal.pone.0253985 (PMC8270166; doi:10.1371/journal.pone.0253985)
Supplement: S3 Table — Log-transformed values of egg characteristic was used as response, and koel vs. host as a fixed factor, to improve normality of residuals (see, S2 Table). (DOCX) [file pone.0253985.s003.docx]

## Supplementary Material

## Table S3: Model outputs from linear mixed-effects models showing differences between Asian koel eggs from specific host nests and their corresponding host eggs (see Figs 2 and 3). Log-transformed values of egg characteristic was used as response, and koel vs. host as a fixed factor, to improve normality of residuals (see, Table S2).

| **Egg character** | **Asian koel vs. host** | ***n***  ***eggs tot*** | ***n clutches*** | ***n***  ***koel eggs*** | ***Estimate host difference*** | ***z*** | ***P*** |  |
| --- | --- | --- | --- | --- | --- | --- | --- | --- |
| Volume | Common Myna | 556 | 295 | 167 | -0.237±0.014 | -16.70 | < 0.001 | *** |
| Volume | House Crow | 326 | 121 | 22 | 0.488±0.030 | 16.19 | < 0.001 | *** |
| Volume | Long-tailed Shrike | 294 | 141 | 82 | -0.804±0.015 | -55.30 | < 0.001 | *** |
| Shape | Common Myna | 556 | 295 | 167 | 0.022±0.006 | 3.86 | < 0.001 | *** |
| Shape | House Crow | 326 | 121 | 22 | 0.077±0.013 | 6.06 | < 0.001 | *** |
| Shape | Long-tailed Shrike | 294 | 141 | 82 | -0.047±0.007 | -6.78 | < 0.001 | *** |
| Max Energy | House Crow | 122 | 47 | 12 | 0.875±0.072 | 12.24 | < 0.001 | *** |
| Max Energy | Long-tailed Shrike | 90 | 49 | 32 | 1.526±0.109 | 14.07 | < 0.001 | *** |
| Prop Energy | House Crow | 122 | 47 | 12 | -0.042±0.049 | -0.87 | 0.387 |  |
| Prop Energy | Long-tailed Shrike | 90 | 49 | 32 | 0.247±0.034 | 7.34 | < 0.001 | *** |
| Sum Energy | House Crow | 122 | 47 | 12 | 0.908±0.075 | 12.12 | < 0.001 | *** |
| Sum Energy | Long-tailed Shrike | 90 | 49 | 32 | 1.266±0.103 | 12.30 | < 0.001 | *** |
| SD Energy | House Crow | 122 | 47 | 12 | 0.875±0.075 | 11.71 | < 0.001 | *** |
| SD Energy | Long-tailed Shrike | 90 | 49 | 32 | 1.518±0.111 | 13.73 | < 0.001 | *** |
