# Supplementary material for: No evidence of host-specific egg mimicry in Asian koels
Source: PLoS One. 2021 Jul 9;16(7):e0253985. doi: 10.1371/journal.pone.0253985 (PMC8270166; doi:10.1371/journal.pone.0253985)
Supplement: S4 Table — P-values were adjusted (P.adj.) following Benjamini and Hochberg. Significant differences are denoted with letters in Figs 4 and 5. (DOCX) [file pone.0253985.s004.docx]

## Supplementary Material

## Table S4: Model outputs from Dunn post host tests, showing pair-wise comparisons of differences in JNDs between Asian koel eggs in common myna nests (egg=AK_CM) and long-tailed shrike nests (egg=AK_LTS). P-values were adjusted (P.adj.) following Benjamini and Hochberg. Significant differences are denoted with letters in Fig. 4 and 5.

| **Egg** | **Visual sys** | **Region** | **Type** | **Pair-egg1** | **Pair-egg2** | **Z** | **P.unadj** | **P.adj** |
| --- | --- | --- | --- | --- | --- | --- | --- | --- |
| AK_CM | UVS | B | Achrom | AK_LTS | CM | -3,207 | 0,001 | 0,008 |
| AK_CM | UVS | B | Achrom | AK_LTS | HC | -2,245 | 0,025 | 0,050 |
| AK_CM | UVS | B | Achrom | CM | HC | 0,962 | 0,336 | 0,504 |
| AK_CM | UVS | B | Achrom | AK_LTS | LTS | -2,566 | 0,010 | 0,031 |
| AK_CM | UVS | B | Achrom | CM | LTS | 0,641 | 0,521 | 0,625 |
| AK_CM | UVS | B | Achrom | HC | LTS | -0,321 | 0,748 | 0,748 |
| AK_CM | UVS | B | Chrom | AK_LTS | CM | -3,421 | 0,001 | 0,004 |
| AK_CM | UVS | B | Chrom | AK_LTS | HC | -2,780 | 0,005 | 0,016 |
| AK_CM | UVS | B | Chrom | CM | HC | 0,641 | 0,521 | 0,521 |
| AK_CM | UVS | B | Chrom | AK_LTS | LTS | -1,604 | 0,109 | 0,163 |
| AK_CM | UVS | B | Chrom | CM | LTS | 1,817 | 0,069 | 0,138 |
| AK_CM | UVS | B | Chrom | HC | LTS | 1,176 | 0,240 | 0,288 |
| AK_CM | VS | B | Achrom | AK_LTS | CM | -3,100 | 0,002 | 0,012 |
| AK_CM | VS | B | Achrom | AK_LTS | HC | -2,298 | 0,022 | 0,043 |
| AK_CM | VS | B | Achrom | CM | HC | 0,802 | 0,423 | 0,634 |
| AK_CM | VS | B | Achrom | AK_LTS | LTS | -2,619 | 0,009 | 0,026 |
| AK_CM | VS | B | Achrom | CM | LTS | 0,481 | 0,630 | 0,757 |
| AK_CM | VS | B | Achrom | HC | LTS | -0,321 | 0,748 | 0,748 |
| AK_CM | VS | B | Chrom | AK_LTS | CM | -3,421 | 0,001 | 0,004 |
| AK_CM | VS | B | Chrom | AK_LTS | HC | -2,298 | 0,022 | 0,043 |
| AK_CM | VS | B | Chrom | CM | HC | 1,122 | 0,262 | 0,314 |
| AK_CM | VS | B | Chrom | AK_LTS | LTS | -0,802 | 0,423 | 0,423 |
| AK_CM | VS | B | Chrom | CM | LTS | 2,619 | 0,009 | 0,026 |
| AK_CM | VS | B | Chrom | HC | LTS | 1,497 | 0,134 | 0,202 |
| AK_CM | UVS | M | Achrom | AK_LTS | CM | -2,138 | 0,033 | 0,065 |
| AK_CM | UVS | M | Achrom | AK_LTS | HC | -1,871 | 0,061 | 0,074 |
| AK_CM | UVS | M | Achrom | CM | HC | 0,267 | 0,789 | 0,789 |
| AK_CM | UVS | M | Achrom | AK_LTS | LTS | -4,009 | 0,000 | 0,000 |
| AK_CM | UVS | M | Achrom | CM | LTS | -1,871 | 0,061 | 0,092 |
| AK_CM | UVS | M | Achrom | HC | LTS | -2,138 | 0,033 | 0,098 |
| AK_CM | UVS | M | Chrom | AK_LTS | CM | -3,474 | 0,001 | 0,003 |
| AK_CM | UVS | M | Chrom | AK_LTS | HC | -2,459 | 0,014 | 0,042 |
| AK_CM | UVS | M | Chrom | CM | HC | 1,016 | 0,310 | 0,372 |
| AK_CM | UVS | M | Chrom | AK_LTS | LTS | -2,085 | 0,037 | 0,074 |
| AK_CM | UVS | M | Chrom | CM | LTS | 1,390 | 0,165 | 0,247 |
| AK_CM | UVS | M | Chrom | HC | LTS | 0,374 | 0,708 | 0,708 |
| AK_CM | VS | M | Achrom | AK_LTS | CM | -2,138 | 0,033 | 0,065 |
| AK_CM | VS | M | Achrom | AK_LTS | HC | -1,871 | 0,061 | 0,074 |
| AK_CM | VS | M | Achrom | CM | HC | 0,267 | 0,789 | 0,789 |
| AK_CM | VS | M | Achrom | AK_LTS | LTS | -4,009 | 0,000 | 0,000 |
| AK_CM | VS | M | Achrom | CM | LTS | -1,871 | 0,061 | 0,092 |
| AK_CM | VS | M | Achrom | HC | LTS | -2,138 | 0,033 | 0,098 |
| AK_CM | VS | M | Chrom | AK_LTS | CM | -3,261 | 0,001 | 0,007 |
| AK_CM | VS | M | Chrom | AK_LTS | HC | -1,871 | 0,061 | 0,123 |
| AK_CM | VS | M | Chrom | CM | HC | 1,390 | 0,165 | 0,247 |
| AK_CM | VS | M | Chrom | AK_LTS | LTS | -0,748 | 0,454 | 0,454 |
| AK_CM | VS | M | Chrom | CM | LTS | 2,512 | 0,012 | 0,036 |
| AK_CM | VS | M | Chrom | HC | LTS | 1,122 | 0,262 | 0,314 |
| AK_CM | UVS | T | Achrom | AK_LTS | CM | -2,352 | 0,019 | 0,056 |
| AK_CM | UVS | T | Achrom | AK_LTS | HC | -1,710 | 0,087 | 0,131 |
| AK_CM | UVS | T | Achrom | CM | HC | 0,641 | 0,521 | 0,521 |
| AK_CM | UVS | T | Achrom | AK_LTS | LTS | -3,955 | 0,000 | 0,000 |
| AK_CM | UVS | T | Achrom | CM | LTS | -1,604 | 0,109 | 0,131 |
| AK_CM | UVS | T | Achrom | HC | LTS | -2,245 | 0,025 | 0,050 |
| AK_CM | UVS | T | Chrom | AK_LTS | CM | -2,459 | 0,014 | 0,028 |
| AK_CM | UVS | T | Chrom | AK_LTS | HC | -2,726 | 0,006 | 0,019 |
| AK_CM | UVS | T | Chrom | CM | HC | -0,267 | 0,789 | 0,947 |
| AK_CM | UVS | T | Chrom | AK_LTS | LTS | -2,833 | 0,005 | 0,028 |
| AK_CM | UVS | T | Chrom | CM | LTS | -0,374 | 0,708 | 1,000 |
| AK_CM | UVS | T | Chrom | HC | LTS | -0,107 | 0,915 | 0,915 |
| AK_CM | VS | T | Achrom | AK_LTS | CM | -2,298 | 0,022 | 0,043 |
| AK_CM | VS | T | Achrom | AK_LTS | HC | -1,710 | 0,087 | 0,105 |
| AK_CM | VS | T | Achrom | CM | HC | 0,588 | 0,557 | 0,557 |
| AK_CM | VS | T | Achrom | AK_LTS | LTS | -4,009 | 0,000 | 0,000 |
| AK_CM | VS | T | Achrom | CM | LTS | -1,710 | 0,087 | 0,131 |
| AK_CM | VS | T | Achrom | HC | LTS | -2,298 | 0,022 | 0,065 |
| AK_CM | VS | T | Chrom | AK_LTS | CM | -3,367 | 0,001 | 0,005 |
| AK_CM | VS | T | Chrom | AK_LTS | HC | -2,245 | 0,025 | 0,050 |
| AK_CM | VS | T | Chrom | CM | HC | 1,122 | 0,262 | 0,314 |
| AK_CM | VS | T | Chrom | AK_LTS | LTS | -0,909 | 0,364 | 0,364 |
| AK_CM | VS | T | Chrom | CM | LTS | 2,459 | 0,014 | 0,042 |
| AK_CM | VS | T | Chrom | HC | LTS | 1,336 | 0,181 | 0,272 |
| AK_LTS | UVS | B | Achrom | AK_CM | CM | -5,450 | 0,000 | 0,000 |
| AK_LTS | UVS | B | Achrom | AK_CM | HC | -4,472 | 0,000 | 0,000 |
| AK_LTS | UVS | B | Achrom | CM | HC | 0,978 | 0,328 | 0,492 |
| AK_LTS | UVS | B | Achrom | AK_CM | LTS | -4,662 | 0,000 | 0,000 |
| AK_LTS | UVS | B | Achrom | CM | LTS | 0,788 | 0,431 | 0,517 |
| AK_LTS | UVS | B | Achrom | HC | LTS | -0,190 | 0,849 | 0,849 |
| AK_LTS | UVS | B | Chrom | AK_CM | CM | -6,399 | 0,000 | 0,000 |
| AK_LTS | UVS | B | Chrom | AK_CM | HC | -4,909 | 0,000 | 0,000 |
| AK_LTS | UVS | B | Chrom | CM | HC | 1,491 | 0,136 | 0,136 |
| AK_LTS | UVS | B | Chrom | AK_CM | LTS | -2,972 | 0,003 | 0,004 |
| AK_LTS | UVS | B | Chrom | CM | LTS | 3,427 | 0,001 | 0,001 |
| AK_LTS | UVS | B | Chrom | HC | LTS | 1,937 | 0,053 | 0,063 |
| AK_LTS | VS | B | Achrom | AK_CM | CM | -5,450 | 0,000 | 0,000 |
| AK_LTS | VS | B | Achrom | AK_CM | HC | -4,424 | 0,000 | 0,000 |
| AK_LTS | VS | B | Achrom | CM | HC | 1,025 | 0,305 | 0,458 |
| AK_LTS | VS | B | Achrom | AK_CM | LTS | -4,709 | 0,000 | 0,000 |
| AK_LTS | VS | B | Achrom | CM | LTS | 0,741 | 0,459 | 0,551 |
| AK_LTS | VS | B | Achrom | HC | LTS | -0,285 | 0,776 | 0,776 |
| AK_LTS | VS | B | Chrom | AK_CM | CM | -6,304 | 0,000 | 0,000 |
| AK_LTS | VS | B | Chrom | AK_CM | HC | -4,462 | 0,000 | 0,000 |
| AK_LTS | VS | B | Chrom | CM | HC | 1,842 | 0,065 | 0,065 |
| AK_LTS | VS | B | Chrom | AK_CM | LTS | -1,994 | 0,046 | 0,055 |
| AK_LTS | VS | B | Chrom | CM | LTS | 4,310 | 0,000 | 0,000 |
| AK_LTS | VS | B | Chrom | HC | LTS | 2,469 | 0,014 | 0,020 |
| AK_LTS | UVS | M | Achrom | AK_CM | CM | -4,140 | 0,000 | 0,000 |
| AK_LTS | UVS | M | Achrom | AK_CM | HC | -3,741 | 0,000 | 0,000 |
| AK_LTS | UVS | M | Achrom | CM | HC | 0,399 | 0,690 | 0,690 |
| AK_LTS | UVS | M | Achrom | AK_CM | LTS | -6,703 | 0,000 | 0,000 |
| AK_LTS | UVS | M | Achrom | CM | LTS | -2,563 | 0,010 | 0,012 |
| AK_LTS | UVS | M | Achrom | HC | LTS | -2,962 | 0,003 | 0,005 |
| AK_LTS | UVS | M | Chrom | AK_CM | CM | -5,611 | 0,000 | 0,000 |
| AK_LTS | UVS | M | Chrom | AK_CM | HC | -4,548 | 0,000 | 0,000 |
| AK_LTS | UVS | M | Chrom | CM | HC | 1,063 | 0,288 | 0,288 |
| AK_LTS | UVS | M | Chrom | AK_CM | LTS | -3,247 | 0,001 | 0,002 |
| AK_LTS | UVS | M | Chrom | CM | LTS | 2,364 | 0,018 | 0,027 |
| AK_LTS | UVS | M | Chrom | HC | LTS | 1,301 | 0,193 | 0,232 |
| AK_LTS | VS | M | Achrom | AK_CM | CM | -4,111 | 0,000 | 0,000 |
| AK_LTS | VS | M | Achrom | AK_CM | HC | -3,731 | 0,000 | 0,000 |
| AK_LTS | VS | M | Achrom | CM | HC | 0,380 | 0,704 | 0,704 |
| AK_LTS | VS | M | Achrom | AK_CM | LTS | -6,741 | 0,000 | 0,000 |
| AK_LTS | VS | M | Achrom | CM | LTS | -2,630 | 0,009 | 0,010 |
| AK_LTS | VS | M | Achrom | HC | LTS | -3,010 | 0,003 | 0,004 |
| AK_LTS | VS | M | Chrom | AK_CM | CM | -5,383 | 0,000 | 0,000 |
| AK_LTS | VS | M | Chrom | AK_CM | HC | -3,845 | 0,000 | 0,000 |
| AK_LTS | VS | M | Chrom | CM | HC | 1,538 | 0,124 | 0,149 |
| AK_LTS | VS | M | Chrom | AK_CM | LTS | -2,393 | 0,017 | 0,025 |
| AK_LTS | VS | M | Chrom | CM | LTS | 2,991 | 0,003 | 0,006 |
| AK_LTS | VS | M | Chrom | HC | LTS | 1,453 | 0,146 | 0,146 |
| AK_LTS | UVS | T | Achrom | AK_CM | CM | -4,244 | 0,000 | 0,000 |
| AK_LTS | UVS | T | Achrom | AK_CM | HC | -3,807 | 0,000 | 0,000 |
| AK_LTS | UVS | T | Achrom | CM | HC | 0,437 | 0,662 | 0,662 |
| AK_LTS | UVS | T | Achrom | AK_CM | LTS | -6,532 | 0,000 | 0,000 |
| AK_LTS | UVS | T | Achrom | CM | LTS | -2,288 | 0,022 | 0,027 |
| AK_LTS | UVS | T | Achrom | HC | LTS | -2,725 | 0,006 | 0,010 |
| AK_LTS | UVS | T | Chrom | AK_CM | CM | -4,519 | 0,000 | 0,000 |
| AK_LTS | UVS | T | Chrom | AK_CM | HC | -4,149 | 0,000 | 0,000 |
| AK_LTS | UVS | T | Chrom | CM | HC | 0,370 | 0,711 | 0,711 |
| AK_LTS | UVS | T | Chrom | AK_CM | LTS | -3,484 | 0,000 | 0,001 |
| AK_LTS | UVS | T | Chrom | CM | LTS | 1,035 | 0,301 | 0,451 |
| AK_LTS | UVS | T | Chrom | HC | LTS | 0,665 | 0,506 | 0,608 |
| AK_LTS | VS | T | Achrom | AK_CM | CM | -4,244 | 0,000 | 0,000 |
| AK_LTS | VS | T | Achrom | AK_CM | HC | -3,760 | 0,000 | 0,000 |
| AK_LTS | VS | T | Achrom | CM | HC | 0,484 | 0,628 | 0,628 |
| AK_LTS | VS | T | Achrom | AK_CM | LTS | -6,580 | 0,000 | 0,000 |
| AK_LTS | VS | T | Achrom | CM | LTS | -2,336 | 0,020 | 0,023 |
| AK_LTS | VS | T | Achrom | HC | LTS | -2,820 | 0,005 | 0,007 |
| AK_LTS | VS | T | Chrom | AK_CM | CM | -4,804 | 0,000 | 0,000 |
| AK_LTS | VS | T | Chrom | AK_CM | HC | -3,570 | 0,000 | 0,001 |
| AK_LTS | VS | T | Chrom | CM | HC | 1,234 | 0,217 | 0,217 |
| AK_LTS | VS | T | Chrom | AK_CM | LTS | -2,146 | 0,032 | 0,048 |
| AK_LTS | VS | T | Chrom | CM | LTS | 2,658 | 0,008 | 0,016 |
| AK_LTS | VS | T | Chrom | HC | LTS | 1,424 | 0,154 | 0,185 |
