# Supplementary material for: No evidence of host-specific egg mimicry in Asian koels
Source: PLoS One. 2021 Jul 9;16(7):e0253985. doi: 10.1371/journal.pone.0253985 (PMC8270166; doi:10.1371/journal.pone.0253985)
Supplement: Attachment — Submitted filename: Nahid et al_Response to reviewer_For Resubmission2.docx [file pone.0253985.s010.docx]

Dear Editor,

Thank you for the opportunity to resubmit this manuscript, PONE-D-19-31106, “No evidence of host-specific egg mimicry in Asian koels Eudynamys scolopaceus” to PLoS ONE. We have made substantial statistical changes throughout the manuscript in response to reviewers’ critique. Importantly, our previous findings of the lack of mimicry are still robust. Below you can find each question from the reviewers in italics, followed by our detailed response, accompanied by references to line numbers for the corresponding changes in the manuscript.

We hope you will consider this revised version of the manuscript for publication in PLoS ONE.

Yours sincerely,

Dr. M. I. Nahid
On behalf of all co-authors

**Response to “Comments to the Author”**

Reviewer #1:

I thank authors for their thorough revision of the manuscript. They dealt with all my comments well. I have only minor comment now.

***Q1:*** *Line 444: here, you cited 101, but it is not a correct reference. The same team of authors deals with the choosing behaviour of the common cuckoo during egg removal in another study. You should cite* *Šulc et al. 2016, Behav. Evol. 27 (1642–1649) (https://doi.org/10.1093/beheco/arw085)*
**Response:** We have changed to correct citation. See line 419.

***Q2*** *Figure 2: I think, Max power should be renamed to „Max energy“ because you use this term throughout the whole manuscript*
**Response:** We agree and have changed this accordingly. See lines 184-185 and Fig. 3.

*Reviewer #2: The authors completely misunderstood my suggestions. Therefore, nothing of the manuscript was improved in terms of statistical analysis. It was rather worsened.*

***Q1*** *The basic model structure should be as the following, where ui indicates egg identity in this case: any egg character ~ species + ui
This model can stand when samples are collected from the same individuals, the same plots, or the same areas repeatedly, meaning that the dataset is longitudinal. However, one can only do such an analysis using a mixed-effect model, because the model has a fixed effect (i.e., species) and a random effect (ui) at the same time. In a longitudinal dataset, each datum is not independent of each other, because some of data are collected from the same egg (i.e., dependent on egg identity), in this case. Ignoring such a difference, i.e., egg identity, is so-called the violation of statistical independence, or pseudo-replication. Most non-parametric tests are not applicable to longitudinal datasets, because they are not designed to do such an analysis. The model structure in most non-parametric tests is the following one:
any egg character ~ species,
and thus applying non-parametric tests to a longitudinal dataset automatically results in pseudo-replication. This is what the authors did by adopting the Mann-Whitney test. Therefore, as long as the authors insist with non-parametric tests to analyse their datasets, the authors should always violate the statistical independence of data.*
**Response:** We made considerable changes throughout the manuscript accordingly.
Originally, we selected non-parametric tests, due to residuals from some linear mixed-effects models slightly violating the assumption of being normally distributed, see revised Table 1. We have now performed an additional set of analyses, using the same models, but using log-transformed values, ensuring normality of the residuals, see the new supplementary table (Table S1). One model still has residuals slightly deviating from a normal distribution, the model for host egg volume. We have indicated this in the manuscript (see lines 226-236), and importantly this marginal violation of model assumptions does not affect these results, where there are large differences among host eggs in egg volume, see Fig. 1.

Importantly, in all models using this model structure, we are now avoiding the issue of non-independence, by instead running a mixed-effects models accounting for e.g. nest/clutch identity for host eggs in the first part of the manuscript, and in the cone stimulation analyses accounting for egg identity.

***Q2*** *There should be another structure in the dataset, which has been overlooked: similarity of eggs within the same nest. Although I pointed out this problem in the first review, the authors did nothing. Egg characteristics are maternal traits, and thus eggs that were laid by a single female should be similar to each other. Therefore, assuming that there is no conspecific brood parasitism in all host species, host eggs from the same nests should be similar to each other. This can be avoided by assigning nest identity nested within species as a random effect. Nest identity should be nested within species not to treat koel eggs differently from host eggs sharing the same nests.*
**Response:** We agree and have changed the models accordingly. All models assessing variation among host eggs have now got the following structure:
response (e.g. volume, shape or pattern variable) ~ egg type + (1|Year) + (1|Nest identity)
see changes in lines 226-236, S1 Table.


***Q3*** *Another problem is added in the revised version, unfortunately. The authors added mentions about log-transformation of data. Log-transformation of data used to be used widely, but nowadays, it is known to be wrong. By using log-transformation, variances are reduced particularly when the raw values are large, leading to underestimation of the variation of data. Thus, differences detected by linear models through log-transformation can be an overestimation. To avoid this, one can adopt a generalised linear model. Since generalised linear models are almost equally easily available to linear models, there should be no reason not to use them.*
**Response:** We agree with this critique to some extent, and have therefore changed this, using un-transformed values in all models. However, to satisfy model assumptions of linear mixed-effects models, namely normally distributed residuals, we ran an additional set of analyses with log-transformed values. Note that these models gave qualitatively similar results, with two exceptions, a difference in max energy between koel eggs from common myna and house crow nests, and a difference in SD energy between koel eggs from house crow and long-tailed shrike nests. None of these relationships seem to be a result of mimicry towards the corresponding host, thus we discuss this in lines 283-293. See also Fig.3 where significant tests using log-transformed data only, are denoted with a footnote.

We have chosen to keep the linear mixed-effects models, where we assess whether our results remain when using log-transformed data (see Table S1). This is a robust procedure, as any underestimation of variation would be detected using both sets of analyses.
Importantly, the main findings and conclusions from this study does not rely on log-transformed data alone. Instead, our findings using un-transformed data are robust, and supporting analyses using log-transformed data corroborates these findings.


Reviewer #3: Minor comments:
Title: yes, this is more in accordance with your data
***Q1*** *Line 5: superscript "1" missing*
**Response:** superscript “1” is now added.

***Q2*** *Lines 89-92: Good that you have included information on Pacific Koel and Long-tailed Cuckoo here as phylogenetic comparison. Though worth noting that Pacific Koel has been selected for host-specific mimicry in another direction so may not tell you much about ancestral state of eggs in Asian Koels.*
**Response:** We have now added that Pacific koel may be an inadequate control for ancestral egg state, see lines 91-96.

Results and Discussion: These are now much clearer having used sub-headings.

***Q3*** *Lines 447-448: This sentence is unclear. Are you making a general statement about brood parasites with egg mimicry here?*
**Response:** Yes and we have revised the line to make it clear, see lines 422-424.

***Q4*** *Lines 454-457: Thick-billed Cuckoo is not particularly closely related to Asian Koel (beyond both being in the cuckoo family). Not clear what the relevance of drawing the comparison with Thick-billed Cuckoo is here. What insight does this tell us about your system or about egg mimicry generally?*
**Response:** We agree in this critique and have decided to remove it as a whole from the discussion.

***Q5*** *It seems that an important bit of information missing is whether individual female Asian Koels specialise on a single host or if the species is a generalist at the individual level. It would be worthwhile for future work to establish this in order to understand why you find no egg mimicry in this system. Some reference to this should be made in Discussion.*
**Response:** This is an important fundament for our hypothesis. In lines 86-87 in the introduction we state that the information regarding host specificity is currently lacking. We have added a line in the discussion as a potential explanation to why we don’t see mimicry to different host species in our study, see lines 429-438.
